# Comprehensive inventory of true flies (Diptera) at a tropical site

**DOI:** 10.1038/s42003-018-0022-x

**Published:** 2018-03-22

**Authors:** Brian V. Brown, Art Borkent, Peter H. Adler, Dalton de Souza Amorim, Kevin Barber, Daniel Bickel, Stephanie Boucher, Scott E. Brooks, John Burger, Zelia L. Burington, Renato S. Capellari, Daniel N. R. Costa, Jeffrey M. Cumming, Greg Curler, Carl W. Dick, John H. Epler, Eric Fisher, Stephen D. Gaimari, Jon Gelhaus, David A. Grimaldi, John Hash, Martin Hauser, Heikki Hippa, Sergio Ibáñez-Bernal, Mathias Jaschhof, Elena P. Kameneva, Peter H. Kerr, Valery Korneyev, Cheslavo A. Korytkowski, Giar-Ann Kung, Gunnar Mikalsen Kvifte, Owen Lonsdale, Stephen A. Marshall, Wayne Mathis, Verner Michelsen, Stefan Naglis, Allen L. Norrbom, Steven Paiero, Thomas Pape, Alessandre Pereira-Colavite, Marc Pollet, Sabrina Rochefort, Alessandra Rung, Justin B. Runyon, Jade Savage, Vera C. Silva, Bradley J. Sinclair, Jeffrey H. Skevington, John O. Stireman III, John Swann, F. Christian Thompson, Pekka Vilkamaa, Terry Wheeler, Terry Whitworth, Maria Wong, D. Monty Wood, Norman Woodley, Tiffany Yau, Thomas J. Zavortink, Manuel A. Zumbado

**Affiliations:** 1 Entomology Section, Natural History Museum of Los Angeles County, 900 Exposition Boulevard, Los Angeles, CA 90007 USA; 2 Royal British Columbia Museum and the American Museum of Natural History, 691-8th Ave. SE, Salmon Arm, BC V1E 2C2 Canada; 3 Department of Plant and Environmental Sciences, 130 McGinty Court, E-143 Poole Agricultural Center, Clemson University, Clemson, SC 29634-0310 USA; 4 Depto. de Biologia, FFCLRP, Universidade de São Paulo, Av. Bandeirantes 3900, 14.040-901 Ribeirão Preto, SP Brazil; 5 Great Lakes Forestry Centre, Canadian Forest Service, Natural Resources Canada, 1219 Queen St. E., Sault Ste. Marie, Ontario, P6A 2E5 Canada; 6 Australian Museum, 1 William Street, Sydney, NSW 2010 Australia; 7 Department of Natural Resource Sciences, McGill University, Macdonald Campus, Ste-Anne-de-Bellevue, Quebec, H9X 3V9 Canada; 8 Canadian National Collection of Insects, Invertebrate Biodiversity, Agriculture and Agri-Food Canada, K.W. Neatby Building, 960 Carling Avenue, Ottawa, Ontario K1A 0C6 Canada; 9 Department of Biological Sciences, Spaulding Hall, University of New Hampshire, Durham, NH 03824 USA; 10 Department of Biological Sciences, Wright State University, 3640 Colonel Glenn Hwy, Dayton, OH 45431 USA; 11 Instituto Federal do Triângulo Mineiro – Campus Uberaba. Rua João Batista Ribeiro 4000, Distrito Industrial II, 38064-790 Uberaba, Minas Gerais Brazil; 12 Departamento de Zoologia, Universidade Federal do Paraná, Jardim das Américas, 81531-980 Curitiba, Paraná Brazil; 13 Mississippi Entomological Museum, Mississippi State University, 100 Old Highway 12, P.O. Drawer 9775, Mississippi State, MS 39762-9775 USA; 14 Department of Biology, Western Kentucky University, Bowling Green, KY 42101 USA; 15 Integrative Research Center, Field Museum of Natural History, Chicago, IL 60605 USA; 16 Independent Investigator, Crawfordville, FL USA; 17 California State Collection of Arthropods, 2683 Tam O’ Shanter Dr., El Dorado Hills, California, CA 95762 USA; 18 California Department of Food and Agriculture, California State Collection of Arthropods, 3294 Meadowview Rd., Sacramento, CA 95832–1448 USA; 19 The Academy of Natural Sciences of Drexel University, 1900 Ben Franklin Parkway, Philadelphia, PA 19103-1195 USA; 20 American Museum of Natural History, Central Park West at 79th St., New York, NY 10024-5192 USA; 21 Department of Entomology, University of California, Riverside, 900 University Ave., Riverside, CA 92521 USA; 22 Zoological Museum, Biodiversity Unit, FI-20014, University of Turku, Helsinki, Finland; 23 Instituto de Ecología, A.C. (INECOL), Red Ambiente y Sustentabilidad, Carretera Antigua a Coatepec 351, Col El Haya, Xalapa, CP 91070 Veracruz, Mexico; 24 Station Linné, Ölands Skogsby 161, SE-38693 Färjestaden, Sweden; 25 I. I. Schmalhausen Institute of Zoology of the National Academy of Sciences of Ukraine, Bogdan Chmielnicki St. 15, 01030 Kyiv, Ukraine; 26 Universidad de Panama, Panama City, Panama; 27 Department of Natural History, University Museum of Bergen, University of Bergen, P.O. Box 7800, 5040 Bergen, Norway; 28 Agriculture and Agri-Food Canada, 960 Carling Avenue, Ottawa, ON K1A 0C6 Canada; 29 School of Environmental Sciences, University of Guelph, Guelph, ON N1G 2W1 Canada; 30 Department of Entomology, Smithsonian Institution, PO Box 37012, MRC 169, Washington, D.C. 20013-7012 USA; 31 Natural History Museum of Denmark, Universitetsparken 15, DK-2100 Copenhagen, Denmark; 32 Institute of Evolutionary Biology and Environmental Studies, University of Zurich, Winterthurerstrasse 190, CH-8057 Zurich, Switzerland; 33 Systematic Entomology Laboratory, USDA, ARS, c/o National Museum of Natural History, MRC-168, P.O. Box 37012, Washington DC, 20013-7012 USA; 34 Natural History Museum of Denmark, Universitetsparken 15, DK-2100 Copenhagen, Denmark; 35 Departamento de Sistemática e Ecologia, CCEN, Universidade Federal da Paraíba, Castelo Branco, s/n, CEP 58.051-900 João Pessoa/PB Brazil; 36 Research Institute for Nature and Forest (INBO), Kliniekstraat 25, B-1070 Brussels, Belgium; 37 Research Group Terrestrial Ecology (TEREC), Ghent University, K.L.Ledeganckstraat 35, B-9000 Ghent, Belgium; 38 Entomology Unit, Royal Belgian Institute for Natural Sciences (RBINS), Vautierstraat 29, B-1000 Brussels, Belgium; 39 USDA Forest Service, Rocky Mountain Research Station, Forestry Sciences Laboratory, 1648 S. 7th Avenue, Bozeman, MT 59717 USA; 40 Department of Biological Sciences, Bishop’s University, 2600 College Street, Sherbrooke, QC J1M 1Z7 Canada; 41 UNESP - Univ Estadual Paulista, Faculdade de Ciências Agrárias e Veterinárias, Departamento de Morfologia e Fisiologia Animal; Via de Acesso Prof. Paulo Donato Castellane, s/n, 14884-900 Jaboticabal, SP Brazil; 42 Canadian National Collection of Insects & Canadian Food Inspection Agency, OPL-Entomology, K.W. Neatby Bldg., C.E.F., 960 Carling Ave., Ottawa, ON K1A 0C6 Canada; 43 Department of Biological Sciences, University of Calgary, 2500 University Drive NW, Calgary, AB T2N 1N4 Canada; 44 Finnish Museum of Natural History, Zoology Unit, University of Helsinki, Helsinki, FI-00014 Finland; 45 Washington State University, 2533 Inter Avenue, Puyallup, WA 98372 USA; 46 Independent Investigator, Hereford, AZ USA; 47 Bohart Museum of Entomology, University of California, One Shields Avenue, Davis, CA 95616 USA; 48 Instituto Nacional de Biodiversidad (INBio), 22-3100 Santo Domingo, Heredia Costa Rica

## Abstract

Estimations of tropical insect diversity generally suffer from lack of known groups or faunas against which extrapolations can be made, and have seriously underestimated the diversity of some taxa. Here we report the intensive inventory of a four-hectare tropical cloud forest in Costa Rica for one year, which yielded 4332 species of Diptera, providing the first verifiable basis for diversity of a major group of insects at a single site in the tropics. In total 73 families were present, all of which were studied to the species level, providing potentially complete coverage of all families of the order likely to be present at the site. Even so, extrapolations based on our data indicate that with further sampling, the actual total for the site could be closer to 8000 species. Efforts to completely sample a site, although resource-intensive and time-consuming, are needed to better ground estimations of world biodiversity based on limited sampling.

## Abstract (web summary)

Brian Brown et al. report the results of the Zurquí All Diptera Biodiversity Inventory project, one of the largest efforts to date to directly assess species richness of a megadiverse order of insects. The authors identified 41,001 flies to 4332 species, including 73 of the world's 160 Diptera families.

## Introduction

Assessing the richness of the world’s biodiversity has long been a goal of biologists. For many groups of organisms, this richness, whether measured in species, interactions, niches, biomass, morphologies, natural histories, behaviors, or other scientific currency, hinges on the megadiverse tropics, especially the New World tropics, where for many groups the greatest number of the world’s species occur^1–3^. May^4^ provocatively stated that what was needed was “to assemble a team of taxonomists, with a comprehensive range of expertise, and then make a rough list of all the species found in one representative hectare in the tropical rain forest… Until this is done, I will not trust any estimate of the global total of species.” Indeed, estimated numbers of world species have continued to range from stunningly large totals, such as 30–100 million species^5–7^ to more modest numbers of 5–10 million species^8–13^.

Insects make up the bulk of the world’s known species^14^, outside of prokaryotes and fungi, in which species-level assignments are not clear in many taxa. Projections about terrestrial diversity largely hinge on the status of four major groups: Coleoptera (beetles), Diptera (true flies), Hymenoptera (wasps, bees, ants), and Lepidoptera (moths and butterflies). Coleoptera comprise the largest number of named species, but Diptera are possibly the least-studied of these megadiverse groups. Each of the four groups is far more diverse in comparison to more popular and intensively studied vertebrates. For example, the fly family Tipulidae (crane flies) alone includes about the same number of named species as all mammals and birds combined^14^.

For entomologists, the goal of understanding more than a small fragment of tropical diversity has heretofore been largely unattainable, due to the shortage of taxonomists, the dearth of funding, challenging curatorial techniques (such as slide mounting), and the overwhelming numbers of unknown species. Relative to the needs of science, collections are small and limited in scope such that Bickel^15^ found collecting intensity to be more correlated with species richness than any inherent characteristics of collecting sites. The number of well-known mainland tropical areas is virtually none. Instead, numbers of species in tropical inventories are estimated by taking small samples of faunas (often including just a few families), and extrapolating against some putatively known larger group or area. Unfortunately, with such extrapolations, there is little anchoring in verifiable data. Even more poorly known is the range of morphologies, life histories, hosts, parasitoids, and other data that make an inventory more than just a number.

One of the world’s best-known biotas is that of Great Britain, where generations of naturalists have searched the landscape looking for, recording, and classifying their local biodiversity. There, ~24,000 species of insects are found^16^, of which about 7,000 species are Diptera^17^, many more than 4000 Coleoptera. In comparison, for all the research that has been done in the mainland tropics (some islands have been more extensively surveyed), almost no single site has been successfully surveyed by a group of scientists for a major group of biodiversity-rich insects. A single exception is a beetle inventory of Dumoga-Bone National Park in Sulawesi, Indonesia^18^, for which a large effort was made to obtain all species.

In the face of the challenge of understanding tropical diversity of Diptera, a group of dipterists decided to determine all species of Diptera collected from a single site, a cloud forest in Zurquí de Moravia, Costa Rica (hereafter referred to as Zurquí). To make this project feasible, the time scale for collecting (one year), geographical scale (four hectares), and collecting protocols^19^ were strictly constrained.

The ZADBI Zurquí All Diptera Biodiversity Inventory project (http://www.tropicalflies.net/) is the first successful modern effort to directly measure species richness of any megadiverse order of insects in a mainland tropical area. We found 4332 fly species based on direct evidence, which is important for other inferences on conservation of natural tropical and non-tropical environments in the world, adjustments of biodiversity study protocols, and understanding the funding needs for study and estimation of the group worldwide.

## Results

### Number of specimens

The ZADBI collecting effort resulted in 613 sampling events at Zurquí. From the many hundreds of thousands of Diptera collected, 52,947 were available for identification; of these 41,001 were identified. Many thousands of others were examined in alcohol but not identified in the database because they were perceived to be duplicates, or were the wrong sex for identification.

### Number of species

From the 41,001 specimens, 4,332 species of Diptera were identified (Supplementary Data 1). Seventy-three of the world’s approximately 160 families were collected. We suspect that a small number of other families could theoretically be present (e.g. *Platypeza* sp., family Platypezidae were collected at Zurquí in 1995), but in general, most families expected to be present were found and treated.

### Largest families

The most species-rich family at this site was the Cecidomyiidae (gall midges), with 800 species recorded (Table 1). As one of the least-studied families of flies in the world (relative to the number of species present), Cecidomyiidae also contain some of the smallest and most fragile specimens. Great care and special handling were necessary to collect, separate and slide mount these specimens without causing damage that would prevent their identification. Only a small fraction of the enormous amount of material collected by the various traps could be mounted and identified, and further work would undoubtedly yield even more species, perhaps allowing the total to exceed 1000. Cecidomyiidae generally have not been included in inventories that use trapping because previous research on plant-associated species of this family concentrated on obtaining all life stages through rearing^20,21^. The ZADBI project is nearly unique in this regard, as we found that specimens of Cecidomyiidae have abundant, previously unused, structural characters, especially in the male genitalia, that make their identification possible. We object to the idea that alone among the Diptera, this family cannot be identified using morphology; the feasibility of this approach is demonstrated by the 800 species recognized herein.

**Table 1:** Number of species from fifteen most species-rich Diptera families at Zurquí (from the current study)

Family	Number of species
Cecidomyiidae	800
Phoridae	404
Tachinidae	286
Mycetophilidae	268
Tipulidae	225
Drosophilidae	219
Sciaridae	204
Ceratopogonidae	200
Dolichopodidae	178
Psychodidae	171
Chironomidae	138
Muscidae	120
Agromyzidae	117
Lauxaniidae	116
Syrphidae	93

A notable exception to the lack of cecidomyiids in inventories is Hebert et al.^22^, who identified taxa of Canadian insects collected by Malaise trapping using DNA barcodes. They found Cecidomyiidae to be significantly underestimated, and extrapolated a worldwide total of 1.8–2.0 million species in this family alone.

The second most species-rich family was Phoridae, with 407 species (Table 1). Like cecidomyiids, phorids were represented in Malaise trap samples in huge numbers, with 100,000 or more specimens from Malaise trap 1 alone. We were unable to process all of this material, but the 407 species recognized is probably well below the actual total present. The third to seventh largest families were Tachinidae, Mycetophilidae, Tipulidae, Drosophilidae, Sciaridae, and Ceratopogonidae, groups that were also documented as abundant and understudied by Brown^23^ in other Neotropical Malaise trap samples.

**Fig. 1:**
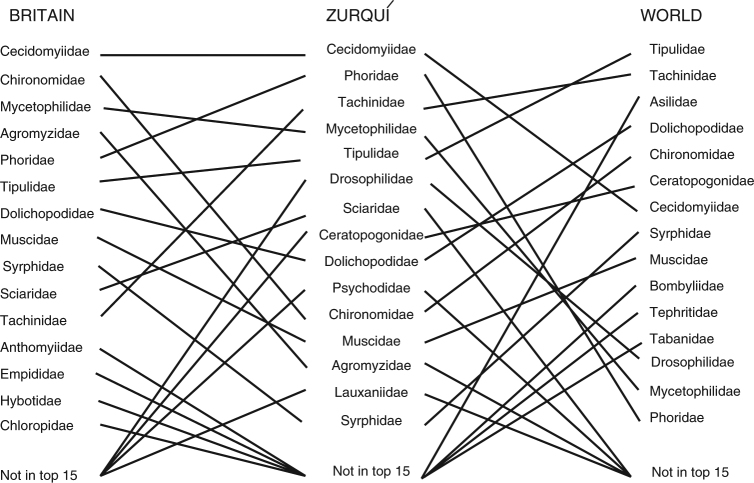
Tanglegram comparison of species richness rankings of top 15 families of Zurquí, Great Britain, and the world. Horizontal lines represent equal ranking; lines sloped downward from Zurquí toward either side join families under-represented relative to Zurquí fauna; lines sloped upwards from Zurquí on either side join families under-represented at Zurquí relative to other faunas

The Zurquí ranking of the top 15 most species-rich families (Table 1) differs from that of the best known fauna, that of Great Britain^17^, and to that of the current world list^24^. This suggests that the current understanding of species richness per family has important biases, and that especially the world list is dominated by the idiosyncratic way that taxonomy develops. Families with large, showy species; those of agricultural, medical, and veterinary importance; and in one case, those that were the subjects of study by a determined and almost superhumanly productive individual (C.P. Alexander describing over 10,000 Tipulidae), led to taxonomic favorites being ranked as most species-rich (Table 2). Our results suggest that the world list in particular is highly deficient in species of Cecidomyiidae, Phoridae, and other taxa whose lines connecting names from Zurquí to the world fauna in Fig. 1 (on the right side) slope downward to the right.

**Table 2:** Fifteen most species-rich Diptera families

Family	Number of described species
Tipulidae s.l.	15,457
Tachinidae	8500
Asilidae	7479
Dolichopodidae	7236
Chironomidae	7054
Ceratopogonidae	6267
Cecidomyiidae	6203
Syrphidae	6016
Muscidae	5210
Bombyliidae	4946
Tephritidae	4911
Tabanidae	4406
Drosophilidae	4315
Mycetophilidae	4164
Phoridae	4105

Numbers are on a worldwide basis^48^, with updated Cecidomyiidae^49^, Ceratopogonidae^50^, Drosophilidae^51^, Tephritidae (compiled by A.L. Norrbom), Tachinidae^52^, and Tipulidae^53^

### Genus-level diversity

Generic concepts are human constructs, but are useful when they represent monophyletic assemblages of species. The overall fauna at Zurquí is not dominated by large radiations of species but instead by many genera with a few species in each. For instance, 49% of the genera were represented by one species, 65% by one or two species. The mean number of species per genus is 4.63. These genera are not restricted to Zurquí; instead, they have relatives in other parts of the world^25,26^, notably other sites in Central America.

In contrast to the overall fauna, two genera are extremely diverse at Zurquí (and elsewhere): *Megaselia* Rondani with 240 species (Phoridae) and *Mycetophila* Meigen with 136 species (Mycetophilidae). Both were considered “open-ended”, nearly impossible groups by Bickel^27^, who declared them too diverse and too difficult to collect in their entirety for us to ever fully know them.

The phorid genus *Megaselia* represents 60% of the phorid fauna at Zurquí (Supplementary Data 1), but with much of the material still not identified due to the overwhelming number of specimens. The taxonomic status of this genus is uncertain because of questions of its monophyly^28–30^. The genus is apparently young (rare or absent from 50 mya Baltic amber), and might represent one of the most enormous terrestrial species radiations of a single genus in the earth’s history.

*Mycetophila* is a morphologically distinctive group, although its monophyly has not been rigorously tested. At Zurquí, this genus is far more diverse than any other mycetophilid genus, making up over half of all species documented in the family (136 of 267; 51%). This proportion is much greater than what is seen in the much better-studied Nordic region of Europe^31–33^, where *Mycetophila* makes up between 9 and 13% of the mycetophilid species. Despite the predominance of *Mycetophila* in tropical habitats, however, only four *Mycetophila* species are named and recorded for all of Costa Rica. It has not yet been ascertained whether any of the four named species were captured at Zurquí or if all 136 species captured in this study are new and undescribed.

### Extrapolations

Based on the Chao 1 formula, our data (*S*_obs_ = 3,925; *#singletons* = 1,777; *#doubletons* = 682) give an estimated total of 6647 species, 1.7 times the observed total. These data exclude the phorids, which were processed differently and all of which are singletons. If phorids were included, and their observed number of species (407) scaled in the same way, the projected number of phorids would be 689 species, and the estimate for the total Diptera fauna would be 7336 for the site.

**Fig. 2:**
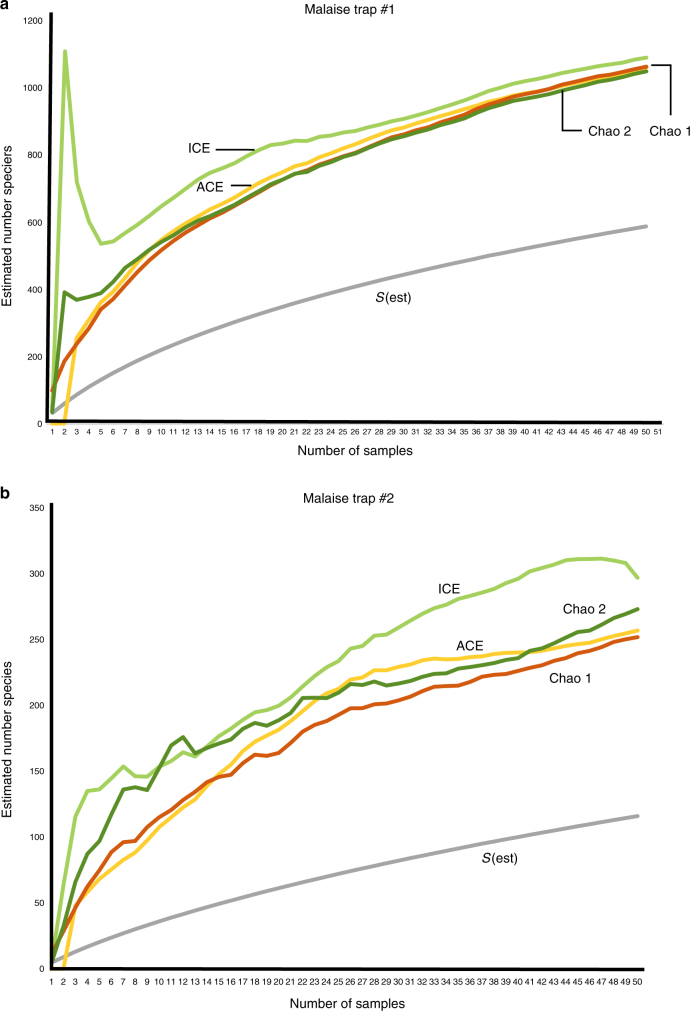
Species accumulation curves for fully extracted and identified families. Estimators from EstimateS:^45^ *S*(est) Expected number of species in *t* pooled samples, given the reference sample(s); ICE Incidence-based Coverage Estimator; ACE Abundance-based Coverage Estimator. **a** Malaise trap 1, **b** Malaise trap 2

**Fig. 3:**
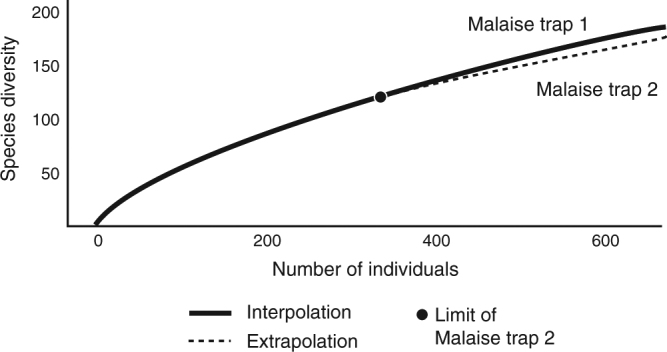
Rarefaction of species accumulation curves for Malaise traps 1 and 2

None of the estimators for the sample-based species accumulation curves for the combined 49 families (for which all specimens were extracted and identified) reached an asymptote, although they began to converge on values (Fig. 2). Both graphs show the number of species accumulated to be well below the expected total, with estimators predicting 1071 species for Malaise trap #1 (1.8 times the 590 species actually collected) and 270 species, for Malaise trap #2 (2.3 times the 116 species actually collected) (Table 3). Rarefaction of the data from the two traps indicate that they were sampling a similar-sized fauna, albeit with Malaise trap #2 collecting more slowly (Fig. 3).

**Table 3:** Results of species accumulation analysis of 49 fully-extracted families of Diptera

	Malaise trap #1	Increase factor	Malaise trap #2	Increase factor
Number of specimens	4752	—	291	—
Observed number of species	590	—	116	—
ACE	1067	1.8	257	2.2
ICE	1095	1.9	297	2.6
Chao 1	1068	1.8	252	2.2
Chao 2	1054	1.8	273	2.4
Mean value	1071	1.8	270	2.3

See Supplementary Data 2 for list

Increase factor=number of species estimated divided by the observed number of species

Estimators from EstimateS:^45^ *S*(est) Expected number of species in *t* pooled samples, given the reference sample(s), *ICE* Incidence-based Coverage Estimator, *ACE* Abundance-based Coverage Estimator

## Discussion

Based on the sample-based estimates from the catch of our two Malaise traps, we found that our collecting underestimates the fauna by at least 1.7 times. Thus, our total of 4332 species could easily be 7,364 species (4332 × 1.7), or more likely even higher, since some of our estimates included only 49 families and did not include, among others, the megadiverse cecidomyiids or phorids. The estimated total is similar to that for the individual-based Chao 1 estimations of 7336 species.

Reasonable and predictive extrapolations of global diversity will become available only when more tropical faunas are interpreted at the species level, including those that are small-bodied or otherwise difficult to curate. Until then, we continue to be highly skeptical of projections, especially those at the lower end of the published ranges. For instance, a study of 0.48 hectares of a lowland tropical forest in Panama^9^ collected 6144 species of arthropods extrapolated using various models to suggest that the total reserve of 6000 hectares supported 25,000 species. Although including 102 researchers to identify an ecologically broad spectrum of taxa, their taxon sampling was actually quite low for the Diptera, which included only four families (compared to the 73 families in the current study). Of these, Dolichopodidae had 132 species, Asilidae 21, Scatopsidae 16 and Stratiomyidae 24 (178, 20, 22, 36 at Zurquí). This led them to suggest the presence of either 1754 or 1429 species of Diptera, depending on global or local comparisons. Even considering their more limited sampling area and different habitat in the lowlands, this contrasts strongly with the ZADBI collection of 4332 species and a total estimation of about 7300 species. It is likely that Diptera were seriously underestimated due to their constricted taxon sampling, much more so than their statement “…if the global species richness of Nematocera is grossly underestimated, then our estimate of total species richness may be more than twice as high as the one reported here…” indicated. It is consequently uncertain what impact this has on their estimation of 25,000 arthropod species present at their site, as well as their larger extrapolated conclusion that “The robust estimates of local arthropod diversity derived in our study thus support previous estimates of global species richness [of 6.1 million species].”

Clearly, the Diptera are much more species-rich than represented in some previous estimates. A comparison of Zurquí diversity^34^ family by family with described species from Central America, Colombia, the entire Neotropical Region and the world, showed that current numbers of named species of Diptera are extremely low. Hebert et al.^22^. extrapolated from their collecting that there might be 50,000 species of Canadian Diptera. Considering the Canadian fauna to be 1% of global biodiversity, their extrapolation would lead to 5 million Diptera species worldwide, a number that is not inconceivable considering the diversity found at Zurquí. In addition, although no other sites have been similarly inventoried, there are indications that Zurquí is not alone in being rich in species. Studies on individual groups of Phoridae, for instance, have uncovered faunas much larger than that of Zurquí, e.g., 127 species of *Apocephalus* Coquillett^35^ and 39 species of *Dohrniphora* Dahl in the lowlands at La Selva, Costa Rica^36,37^, versus 26 *Apocephalus* and 9 *Dohrniphora* at Zurquí.

The only somewhat comparable data from other insect orders is the beetle survey in Indonesia^18^. Given that this survey took place over a much larger area (500 ha), including elevations from 200 to 1140 m, it is difficult to relate their preliminary number of 5649 species to that documented herein for a site two orders of magnitude smaller.

The species accumulation curves and projections (Figs. 2 and 3) for the ZADBI collections indicate that the entire fauna still has been inadequately sampled. Therefore, even with its tremendous number of specimens collected and identified, the ZADBI project fell short of documenting all Diptera of this site. This does not represent a failure of the project, as the ZADBI project has generated a large amount of baseline data for future tropical inventories, but a reflection of the difficulty of doing a proper survey. Reasons for the shortfall might include undersampling of the forest canopy, where much of tropical forest diversity is assumed to exist^9,38,39^, insufficient or incorrect use of some trapping methods, or simply not collecting long enough.

The ZADBI project, although tightly constrained, uncovered 4332 species of Diptera in a mere four hectares of property. These results suggest that conservative estimates of world Diptera biodiversity based on much less thorough sampling are probably too low, not only for their underestimation of megadiverse Cecidomyiidae, but also for the legions of other small-to-large, undescribed flies. Furthermore, a tremendous number of evolutionary lineages are involved in this diversity, with low numbers of species per genus being the norm, indicating that similar diversity will be found elsewhere. Many species are small in size, meaning that fine morphological examination will be necessary to interpret character evolution, even as molecular methods begin to become more common in performing quantitative assessments of biodiversity (e.g., DNA barcoding^22^ or high throughput sequencing after homogenizing samples with a blender^40^). Many more large-scale, intensive inventories at other sites are needed to determine the distribution of this spectacular Diptera diversity and to fully document as much of it as possible before these species disappear. Our protocol and results provide a model for such future inventories.

## Methods

### Sampling protocol

Methods of the ZADBI project were previously outlined in detail^19^, including rationale for selecting this site. In short, Zurquí was selected to maximize biodiversity, based on anecdotal data from collections made at the site over the 20 years since its discovery, and to minimize difficulties, being on private land and located a short distance from the collaborating Instituto Nacional de Biodiversidad (INBIO). A variety of sampling methods were used at a partially disturbed cloud forest site at Zurquí (10.05°N, 84.01°W, 1600 m elevation) for one year. The four-hectare collecting site consisted of two forested ravines and some pasture adjacent to extensive primary forest in Braulio Carrillo National Park (Fig. 1 in Borkent and Brown^19^). We report here primarily on the catch from two Malaise traps (light-weight Townes model^41^, purchased from Sante Traps) that were operated in the same locations throughout the one-year sampling period. Malaise trap 1 was placed near a forest edge, with the area in front of the trap consisting of a rough lawn. Malaise trap 2 was placed in the ravine near the small permanent stream. Three days per month, Malaise trapping was supplemented by sweep netting, flight-interception trapping, light trapping, pan trapping, baiting, and emergence trapping. Three additional Malaise traps were operated to provide a specimen pool for families represented by low numbers. All Diptera were separated, sorted to family, then either curated, or for perceived duplicates of previously extracted species, left in alcohol. Curated specimens were fully prepared to our specifications, generally as dried or slide-mounted specimens, entered into our database, and distributed to each of us for detailed study. Species identifications were based on morphological criteria only. Once identified, names of species, or numbered morphotypes were added to our database (Supplementary Data 1). Although not fully indicative of whether or not a species was previously described, we note that 93% of all species identifications were morphospecies, i.e., not immediately recognized by experts as named taxa. Further taxonomic work, in many cases full revisions, would be necessary to recognize how many undescribed species were present in the material.

Phoridae were treated slightly differently, in that samples were examined and only presumed novel species were removed, recorded, and entered into our database.

### Species number estimates

An estimate of the number of species actually occurring at Zurquí was made using the Chao 1 formula^42,43^, $S_{Chao1} = S_{observed} + (\frac{{\# singletons^2}}{2} \times \# doubletons)$, where *#singletons* is the number of species known from only one specimen and *#doubletons* those only known from two specimens (this formula is only valid if *#doubletons > *0). All families and all samples were used for this estimation, except phorids, whose sorting only recorded singletons. To study the effectiveness of the two primary Malaise traps, sample-based species accumulation curves were developed with EstimateS^44^, using abundance data for 49 families of Diptera (less diverse families that could be fully extracted and identified: Agromyzidae, Anisopodidae, Anthomyiidae, Anthomyzidae, Asilidae, Asteiidae, Aulacigastridae, Bombyliidae, Calliphoridae, Chamaemyiidae, Clusiidae, Conopidae, Ctenostylidae, Culicidae, Diastatidae, Dixidae, Ephydridae, Fanniidae, Heleomyzidae, Inbiomyiidae, Lonchaeidae, Lygistorrhinidae, Micropezidae, Milichiidae, Neriidae, Odiniidae, Oestridae, Periscelididae, Piophilidae, Pipunculidae, Pseudopomyzidae, Pyrgotidae, Rhagionidae, Rhinophoridae, Richardiidae, Sarcophagidae, Scathophagidae, Scatopsidae, Sepsidae, Simuliidae, Stratiomyidae, Syrphidae, Tabanidae, Tachinidae, Tanypezidae, Tephritidae, Ulidiidae, Xylomyidae, Xylophagidae) from 50 week-long Malaise trap samples from Malaise traps 1 and 2. The program considers each sample to be a unit for constructing a species accumulation curve, randomizes the samples and smooths the curves based on sampling within all samples (Supplementary Data 2). Rarefaction of the two sample sets was performed using iNext Online;^45^ this procedure compares samples for distinct sites and equalizes the sampling effort based on the number of specimens, rather than samples (Supplementary Data 3). The point at which the samples are joined based on the limitation of the number of specimens in the smaller sample, is shown by a large dot in Fig. 3.

Family classification follows that of Brown et al.^46,47^, other than the Empididae *sensu lato*, which are here treated as three families, namely Empididae, Brachystomatidae, and Hybotidae.

### Data availability

The data underlying the species accumulation analyses are given in Supplementary Materials.

## Electronic supplementary material

Description of Additional Supplementary Files(PDF 35 kb)

Supplementary Data 1(XLSX 113 kb)

Supplementary Data 2(XLSX 460 kb)

Supplementary Data 3(XLSX 14 kb)
