# Supplementary material for: Comprehensive inventory of true flies (Diptera) at a tropical site
Source: Commun Biol. 2018 Mar 22;1:21. doi: 10.1038/s42003-018-0022-x (PMC6123690; doi:10.1038/s42003-018-0022-x)
Supplement: Supplementary file 1 — Description of Additional Supplementary Files(PDF 35 kb) [file 42003_2018_22_MOESM1_ESM.pdf]

Description of Additional Supplementary Files

File Name: Supplementary Data 1
Description: List of all species and morphospecies of Diptera identified from Zurquí.

File Name: Supplementary Data 2
Description: Data set to analyze catch of Malaise trap 1 and 2 using EstimateS.

File Name: Supplementary Data 3
Description: Data set for rarefaction analysis using iNextOnline.
